# Disease Risk in Temperate Amphibian Populations Is Higher at Closed-Canopy Sites

**DOI:** 10.1371/journal.pone.0048205

**Published:** 2012-10-31

**Authors:** C. Guilherme Becker, David Rodriguez, Ana V. Longo, Amanda L. Talaba, Kelly R. Zamudio

**Affiliations:** Department of Ecology and Evolutionary Biology, Cornell University, Ithaca, New York, United States of America; Smithsonian's National Zoological Park, United States of America

## Abstract

Habitat loss and chytridiomycosis (a disease caused by the chytrid fungus *Batrachochytrium dendrobatidis* - *Bd*) are major drivers of amphibian declines worldwide. Habitat loss regulates host-pathogen interactions by altering biotic and abiotic factors directly linked to both host and pathogen fitness. Therefore, studies investigating the links between natural vegetation and chytridiomycosis require integrative approaches to control for the multitude of possible interactions of biological and environmental variables in spatial epidemiology. In this study, we quantified *Bd* infection dynamics across a gradient of natural vegetation and microclimates, looking for causal associations between vegetation cover, multiple microclimatic variables, and pathogen prevalence and infection intensity. To minimize the effects of host diversity in our analyses, we sampled amphibian populations in the Adirondack Mountains of New York State, a region with relatively high single-host dominance. We sampled permanent ponds for anurans, focusing on populations of the habitat generalist frog *Lithobates clamitans*, and recorded various biotic and abiotic factors that potentially affect host-pathogen interactions: natural vegetation, canopy density, water temperature, and host population and community attributes. We screened for important explanatory variables of *Bd* infections and used path analyses to statistically test for the strength of cascading effects linking vegetation cover, microclimate, and *Bd* parameters. We found that canopy density, natural vegetation, and daily average water temperature were the best predictors of *Bd*. High canopy density resulted in lower water temperature, which in turn predicted higher *Bd* prevalence and infection intensity. Our results confirm that microclimatic shifts arising from changes in natural vegetation play an important role in *Bd* spatial epidemiology, with areas of closed canopy favoring *Bd*. Given increasing rates of anthropogenic habitat modification and the resulting declines in temperate and tropical frogs, understanding how vegetation cover and disease interact is critical for predicting *Bd* spread and developing appropriate management tools for wild populations.

## Introduction

Anthropogenically driven habitat change has important implications for host-pathogen interactions, because even slight changes in environmental conditions can modify numerous biotic and abiotic factors that influence these interactions [1]–[5]. Habitat modification can alter host-pathogen dynamics by regulating host species richness [1], [6], population size, isolation [7], and inbreeding [8], or by shifting macro [9] and microclimates [10] to conditions detrimental or favorable to hosts or pathogens [11]–[13]. Therefore, studies investigating the links between habitat change and disease require integrative approaches to control for the multitude of possible interactions in spatial epidemiological research [14].

Shifts in microclimate and changes in host community structure across gradients of habitat alteration play important roles in amphibian epidemiology [6], [15]. The frog killing fungus *Batrachochytrium dendrobatidis* (*Bd*), for instance, is more prevalent and occurs at higher infection intensities in pristine tropical forests compared to disturbed habitats [6]. Typically, shade, humidity, and host diversity are higher in natural forests, whereas temperature and host community evenness are often highest in disturbed areas [10], [16], [17]. This pattern holds both for tropical and temperate forests. However, decreases in host diversity and local species turnover along gradients of habitat alteration are often less pronounced in temperate zones [18], [19], despite similar changes in microclimate that result from removal of natural vegetation. Thus, temperate amphibian populations that persist in a mosaic of altered landscapes provide an opportunity to investigate the effects of microclimate on amphibian host-pathogen interactions in the absence of the strong confounding effects of host diversity on disease dynamics. Even though multiple studies have modeled the role of regional and large scale climate in *Bd*-induced amphibian declines [12], [20], [21], we have not yet fully characterized how habitat change affects local microclimate, which may in turn control pathogen infections [22], [23].

From the host's perspective, immune responses usually decrease as a result of the multiple effects of habitat alteration [24], [25]. Microclimatic changes caused by deforestation can shift thermal physiology and hydric conditions beyond tolerance limits of forest-associated amphibians [26], [27]. Because amphibians rely on thermoregulation to maintain homeostasis, changes in temperature and humidity along gradients of natural vegetation can affect their immune responses to pathogens [27], [28]. In addition to temperature variability, exposure to environmental contaminants in disturbed habitats hinders essential components of the host immune system [29]–[31]. Habitat change also increases stress hormone production, therefore decreasing host immune capacity [24] and increasing susceptibility to disease in non-natural environments [25], [29]. From the pathogen's perspective, deforestation can shift air and water temperatures to levels that exceed the upper threshold (25°C) of the optimal microclimatic envelope for *Bd*, thus limiting pathogen growth and persistence [32], [33], [22]. Removal of canopy cover often reduces complexity of aquatic vegetation and of leaf-litter substrates, which might contribute to lower *Bd* persistence in these environments [23]. Therefore, disease risk in amphibian populations will depend on the severity of the environmental change imposed by land-use practices and by the degree to which both hosts and the pathogen respond to the resulting microclimatic changes.

Here, we examined the infection dynamics of *Bd* (prevalence and infection intensity) in populations of the common Green Frog (*Lithobates clamitans*) across a gradient of natural vegetation and microclimate. We sampled frogs in the southern Adirondack Park, New York State, a region with relatively low amphibian diversity and high dominance of this habitat generalist host [34]. Our main goals were to (i) test the hypothesis that natural vegetation surrounding aquatic breeding sites in temperate forests is a significant predictor of *Bd* in amphibians and (ii) test for causal associations linking vegetation cover and microclimate with both *Bd* prevalence and infection intensity. Combined, these goals may elucidate whether vegetation and microclimate modulate disease risk in temperate amphibian populations affected by anthropogenic habitat change.

## Methods

### Study System

We sampled anuran populations in the Adirondack Park of the State of New York (43°27′N, 74°67′W). This region is heavily forested with elements representative of temperate and boreal forests [35], but includes areas with moderate urbanization and agriculture. The fungal pathogen *Bd* is enzootic and widespread in the northeastern U.S. [36], including the Adirondacks. We sampled ten permanent ponds within a period of 15 days to avoid seasonal effects on host behavior and pathogen dynamics. We restricted our sampling to the period of June18th–July 2nd 2011, when environmental temperatures are suitable for *Bd* growth in this region [36]. We recorded all anurans present in our sampling ponds, but focused on the common Green Frog (*Lithobates clamitans*, Ranidae), the locally dominant amphibian host species. Green Frogs breed in permanent ponds during the boreal spring and summer, and typically spend most of the time at the shallow banks of water bodies [37]. They can tolerate a variety of habitats ranging from closed-canopy to open grassland ponds [37], [38]. We conducted diurnal and nocturnal visual encounter surveys around each of our study ponds with a consistent sampling effort of 4.7±0.75 SD hours.person.pond^−1^. We recorded body weight (g) for each captured individual and screened post-metamorphic frogs with sterile swabs to quantify *Bd* prevalence and infection intensity (average of 16.6 *L. clamitans* per site). We tested samples for *Bd* in singlicate using Taqman qPCR [39], [40]; with standards of 0.1, 1, 10, 100, and 1000 zoospore genomic equivalents (g.e.) to determine the presence and infection intensity of *Bd* in each sample. This protocol maximizes amplification efficiencies by diluting extracts to reduce inhibition in environmental samples. For calculations of *Bd* prevalence, we categorized individuals as *Bd*-positive when their qPCR showed an infection load of greater than or equal to one g.e. [39], [40]. We defined *Bd* prevalence as the percentage of infected individuals and *Bd* infection intensity as average number of g.e. per population.

### Biological and Environmental Predictors of Bd

We recorded average host body weight and capture rate (i.e., captured frogs.person.hour^−1^) as a proxy for host population density. We specifically chose this study system because of its relatively low species diversity and high single host species dominance. These community attributes allow us to test hypothesis about the roles of microclimate in disease dynamics without the potential effects of complex host community structure. Nonetheless, we recorded host community diversity (Simpson's *D* [41]) and overall community biomass (i.e., the sum of weights for all captured individuals) for each of our sampling ponds. We assessed natural vegetation cover for each of our 10 sampling sites based on high-resolution orthophotos from 2008–2009 (15 and 30 cm resolution; [42]). For each sampling site, we measured the percentage of natural vegetation cover in a radius of 30 m from the edge of the pond. We considered urban, pasture, agriculture, silviculture, and recreational land (e.g., golf-courses) as non-natural land-cover types. The selected study sites represented a gradient of cover quality ranging from 2 to 95 percent natural vegetation. We chose sites with low topographic variability (mean elevation of sampling sites 471.3±120.79 m SD) to minimize the effects of elevation and macroclimate on host-pathogen dynamics [43], [44]. Using a canopy densiometer in the field, we measured fine scale canopy density (% canopy cover) at 10 m intervals along the water line [45] and averaged these records for each pond. Although we expect that the GIS-based measurements of natural vegetation will be positively correlated with canopy density measured in the field, natural vegetation can vary considerably in height and leaf coverage. Thus, canopy density is a better index of vegetation structure, shade, and understory microclimate. We recorded surface water temperatures (i.e., daily average temp., average daily maximum temp., and average daily minimum temp.) for each sampling pond using waterproof data loggers (Hobo UA-002-64; 0.1°C resolution). We placed one data logger in each pond at 10 cm depth at the shallow margin where amphibian captures were concentrated. We used 30 min interval temperature records taken simultaneously at all ponds for a period of 15 days following completion of sampling at all sites. We collected all environmental and host-pathogen data within a month, minimizing potential seasonality effects [22], [46]. We compared mean air temperatures from Glens Falls, NY, during the host (19.67±1.66°C SD) and environmental sampling periods (21.78±1.72°C SD), and found that the ranges of environmental temperatures highly overlapped during the month-long study period.

### Statistical Analyses

To control for the effects of spatial autocorrelation among ponds, we analyzed our data using conditional autoregressions (CAR). We used CAR to test the relationship of each explanatory variable with *Bd* prevalence or infection intensity. We then used model selection tests including all biological and environmental variables and their interactions to find the combinations of variables that best explained *Bd*. Competing models were ranked based on Akaike Information Criterion (AICc), and we reported the model with the highest goodness-of-fit for each run. We also used CAR to test for associations of natural vegetation with host population (i.e., host average body weight, capture rate) and community attributes (i.e., host community diversity, overall community biomass).

We used path analyses to statistically test for the strength of unidirectional cascading effects linking natural vegetation, canopy density, water temperature, and *Bd* infection parameters. Because canopy density may be a better proxy for microclimate than our actual temperature records from a single data logger per pond, we tested an alternative path diagram in which canopy density directly affected *Bd* infection parameters. We compared goodness-of-fit among models using Expected Cross-validation Index (ECVI), an AIC-based index. We conducted CAR using Spatial Analysis in Macroecology v4.0 [47] and path analyses using Systat v.10.1 [48].

## Results

**Figure 1:**
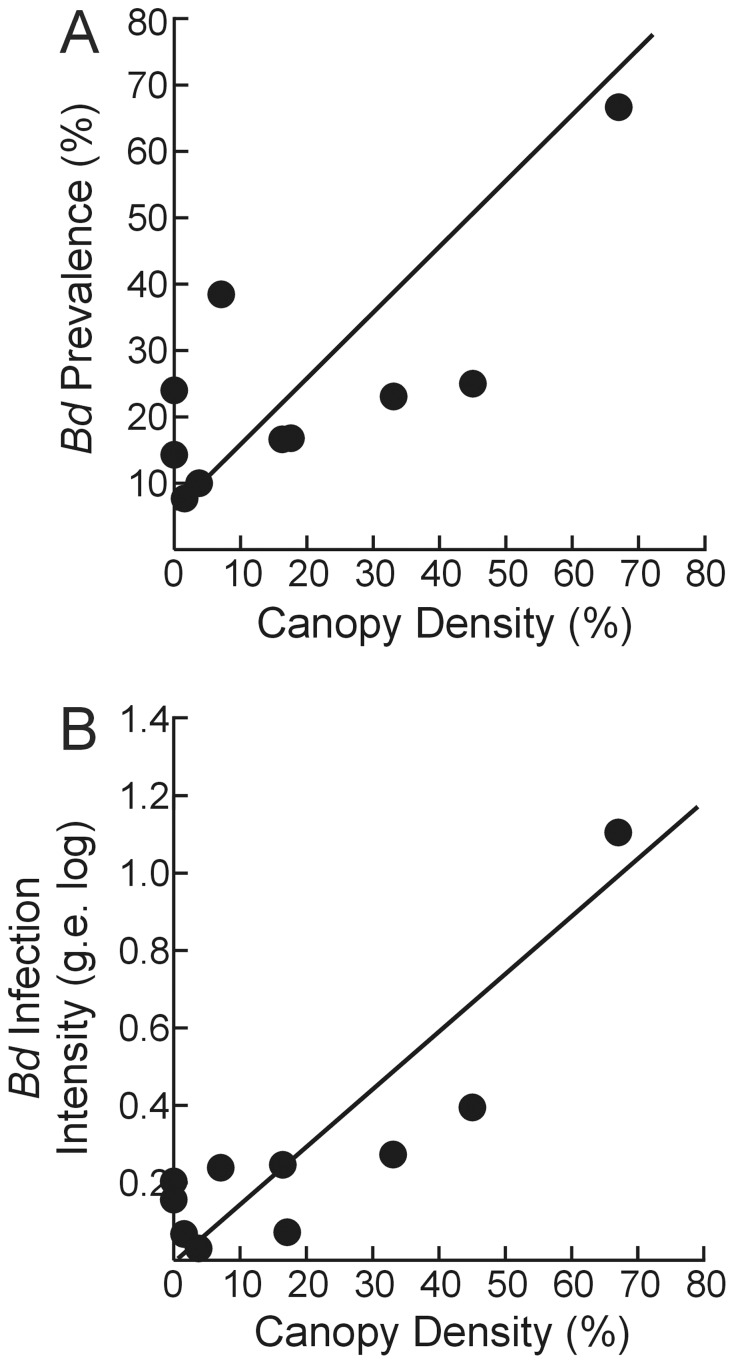
Effect of canopy density on *Bd* across populations of *L. clamitans* from the Adirondack region, New York, USA. (A) *Bd* prevalence; (B) *Bd* infection intensity.

We detected *Bd* at all study sites with mean prevalence of 24.25%±16.41 SD and mean infection intensities of 29.36±151.60 SD, reaching a maximum load of 1063.23 g.e. in our focal species without observed mortalities. Canopy density was the best predictor of *Bd* prevalence in *L. clamitans* (*β*_CAR_ = 0.765, P = 0.001, Fig. 1A), followed by water temperatures [daily average temp. (*β*_CAR_ = −6.125, P = 0.004), maximum daily average temp. (*β*_CAR_ = −5.187, P = 0.004), minimum daily average temp. (*β*_CAR_ = −6.000, P = 0.010)], and natural vegetation (*β*_CAR_ = 0.454, P = 0.021). We found no significant relationships between *Bd* prevalence and average body weight, capture rate, elevation, host community diversity, or overall host community biomass.

We found similar results for *Bd* infection intensity. Canopy density best predicted *Bd* infection intensities (*β*_CAR_ = 0.015, P<0.001, Fig. 1B), with populations in areas of closed-canopy having higher pathogen loads. We found that water temperatures [daily average temp. (*β*_CAR_ = −0.118, P = 0.001), maximum daily average temp. (*β*_CAR_ = −0.097, P = 0.002), minimum daily average temp. (*β*_CAR_ = −0.118, P = 0.003)], natural vegetation (*β*_CAR_ = 0.009, P = 0.007), average body weight (*β*_CAR_ = −0.011, P = 0.019), and capture rate (*β*_CAR_ = 0.110, P = 0.050) were also significant predictors of *Bd* infection intensity in *L. clamitans*. Similar to prevalence, we found no direct associations of *Bd* infection intensity with elevation, host community diversity, or overall host community biomass.

Looking simultaneously at all environmental and biological factors explaining *Bd* prevalence and infection intensity, our model selection identified three key environmental factors: canopy density, natural vegetation, and daily average water temperature (Table S1). The best model explaining *Bd* prevalence included only canopy density as a positive predictor (Table 1). The best model explaining *Bd* infection intensity included natural vegetation as a positive predictor, daily average water temperature as a negative predictor, and the interaction between those two variables (Table 1).

**Table 1:** Conditional autoregressive models (CAR) simultaneously testing the effects of natural vegetation, canopy density, and water temperature on *Bd* prevalence and infection intensity in amphibian populations from the Adirondack region, New York, USA.

Term	*β_CAR_*	*Std. coeff.*	*SE*	*t*	*P*
Prevalence					
*Constant*	*8.966*	*0*	*44.508*	*0.201*	*0.846*
**Canopy density**	**0.765**	**0.999**	**0.143**	**5.363**	**0.001**
Infection Intensity					
*Constant*	*4.189*	*0*	*0.413*	*10.136*	*<.001*
***i)*** ** Natural vegetation**	**0.045**	**5.223**	**0.005**	**8.826**	**<.001**
***ii)*** ** Water temperature - daily average**	**−0.179**	**−1.538**	**0.016**	**−11.066**	**<.001**
***i)×ii)***	**0.002**	**5.592**	**<.001**	**8.300**	**<.001**

Whole-model tests: prevalence: (F = 7.418, n = 10, r^2^ = 0.481, P = 0.026); infection intensity: (F = 38.376, n = 10, r^2^ = 0.950, P<0.001). Std. coeff. stands for standard coefficient. Final models chosen based on Akaike Information Criterion (AICc).

**Figure 2:**
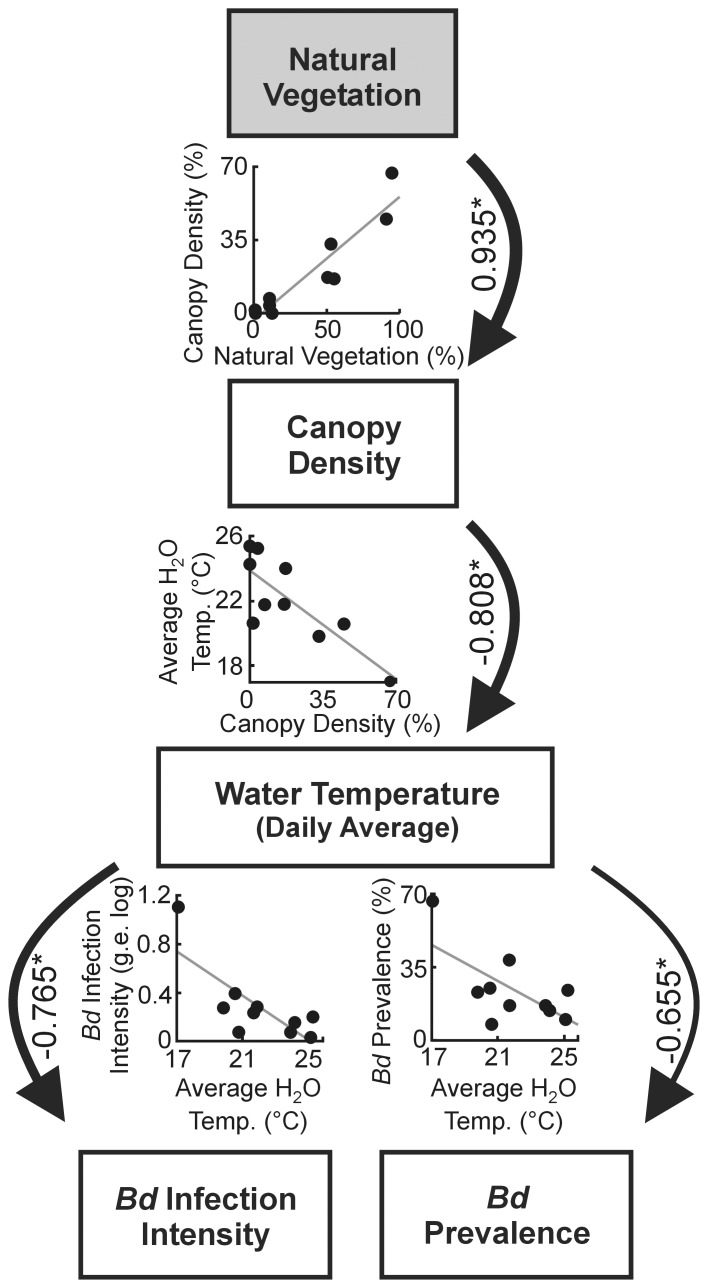
Path analyses indicating a unidirectional causal relationship between natural vegetation, canopy density, water temperature, and *Bd*. The relative strength of each effect is indicated by line width. Linear regressions are shown for each relationship. Numbers are standardized path coefficients (*P<0.05). Diagram shows models for *Bd* prevalence and infection intensity combined.

**Figure 3:**
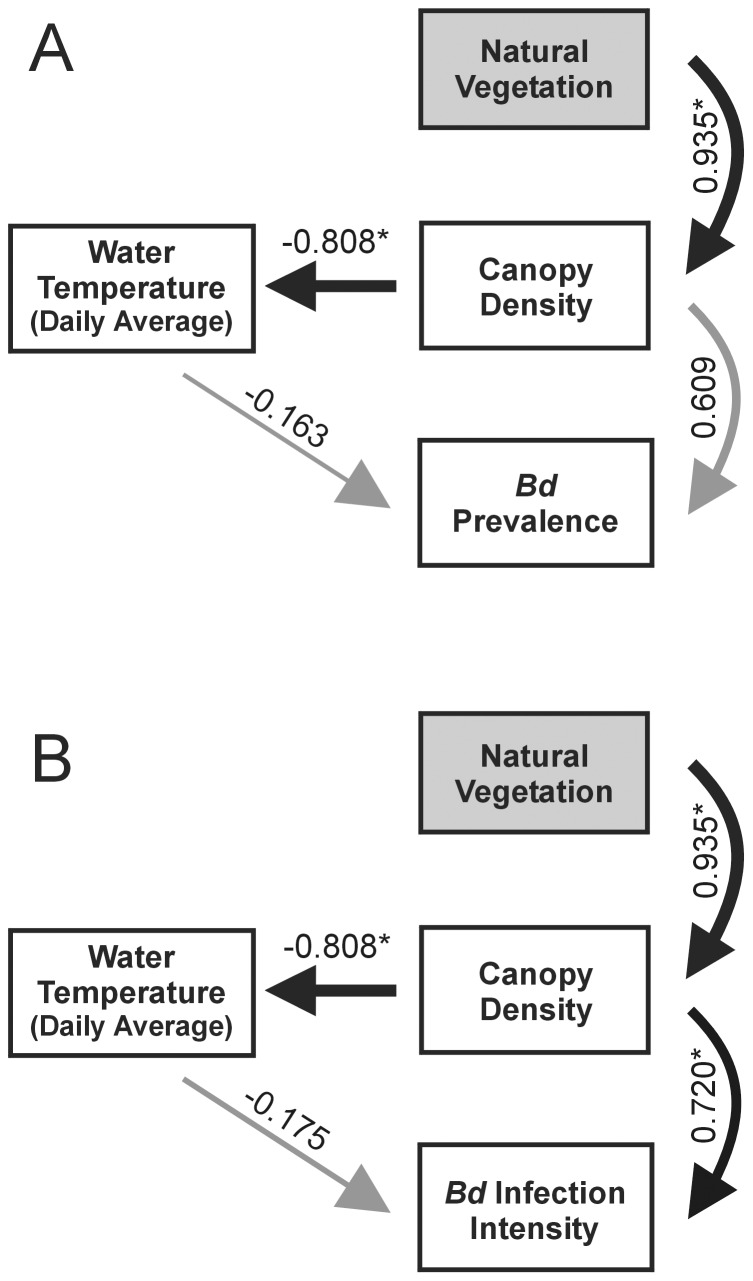
Alternative path models, including both direct and indirect effects of canopy density on *Bd*. (A) *Bd* prevalence; (B) *Bd* infection intensity. The relative strength of each effect is indicated by line width. Grey lines stand for non-significant effects. Numbers are standardized path coefficients (*P<0.05).

We tested for cascading effects among the environmental variables with highest explanatory power for *Bd* infection dynamics (Table 1, Table 2) and found that high canopy density resulted in lower water temperature (i.e., daily average temp.), which in turn predicted higher *Bd* prevalence (ECVI = 2.405; Confidence Interval = 1.90, 4.07) and infection intensity (ECVI = 2.641; CI = 1.90, 4.478; Fig. 2). Thus, habitat change strongly affected patterns of infection dynamics in our temperate amphibian populations, in that frogs in ponds surrounded by natural vegetation showed higher *Bd* prevalence and infection intensity. In the alternative path models, including both direct and indirect effects of canopy density on *Bd* parameters, we found that canopy density was a direct positive predictor of *Bd* infection intensity (ECVI = 2.290; CI = 2.00,3.72; Fig. 3B), but not a significant predictor of *Bd* prevalence (ECVI = 2.371; CI = 2.00, 3.87; Fig. 3A). Both the strictly unidirectional diagram (Fig. 2) and the alternative models including direct effect of canopy density on *Bd* (Fig. 3A, 3B) showed the same goodness-of-fit according to ECVI. These results corroborate earlier findings in both tropical [6] and temperate [23] amphibian populations.

**Table 2:** Best explanatory variables predicting *Bd* prevalence (%) and infection intensity (average g.e. log) across ten sites in the southern Adirondack region, New York, USA [Habitat loss (%), canopy density (%), and average water temperature (°C)].

Site	Lat.	Long.	*Bd* prevalence	*Bd* Infection Int.	Habitat Loss	Canopy Density	Average Water Temp.
1	43.138	−74.921	24.000	0.204	98.394	0	25.377
2	43.060	−74.856	38.462	0.239	88.836	7.083	21.775
3	43.362	−74.587	66.667	1.105	5.269	67.014	17.052
4	43.472	−74.414	7.692	0.068	98.659	1.563	20.635
5	43.016	−74.674	25.000	0.395	9.035	45.023	20.583
6	43.391	−74.719	14.286	0.156	87.066	0	24.259
7	43.390	−74.774	10.000	0.031	88.791	3.750	25.238
8	43.348	−74.617	16.667	0.247	44.683	16.443	21.798
9	43.301	−74.565	16.667	0.072	49.132	17.113	24.002
10	43.392	−74.544	23.077	0.273	46.856	33.073	19.827

The high evenness and low diversity among our sampling ponds (*D* = 0.840±0.23 SD) underscores the dominance of the focal species across this landscape (i.e., *L. clamitans*: N = 166, *L. catesbeianus*: N = 16, *L. palustris*: N = 2, and *L. septentrionalis*: N = 1). The amount of natural vegetation surrounding our sampling ponds did not significantly predict host population and community attributes; namely host average body weight (*β*_CAR_ = 0.344, P = 0.145), host capture rate (*β*_CAR_ = −0.024, P = 0.175), overall host community biomass (*β*_CAR_<0.001, P = 0.119), species richness (*β*_CAR_ = 0.009, P = 0.215), and host diversity (*β*_CAR_ = −0.002, P = 0.399).

## Discussion

The effects of habitat loss and forest fragmentation on host-pathogen interactions are varied [1]–[3], [5], [6], and depending on the mechanisms underlying disease dynamics habitat change can either increase or decrease disease risk. A shift in microclimate is sometimes the leading mechanism controlling disease dynamics [5]. Our data confirm that microclimate shifts arising from disturbance of natural vegetation play an important role in amphibian host-pathogen interactions in temperate systems. Our path analyses underscore the importance of differences in temperature associated with canopy density as a likely driver of amphibian disease risk in natural forests, corroborating a pattern reported earlier in tropical [6] and temperate systems [23]. Host community attributes did not play an important role in our study system, a result that is not surprising given the high dominance of *L. clamitans* and low amphibian diversity of the eastern forest-boreal transition [34], [37]. Our sampling period excluded two local species that breed earlier in the spring when temperatures are cooler (i.e., *Pseudacris crucifer* and *L. sylvaticus*). Although these species may help maintain *Bd* throughout the cold months, it is unlikely that they have a strong effect on disease dynamics later in the season, because they spend long periods of time away from water bodies [49] and breed during periods of suboptimal temperature conditions for *Bd* growth [32]. Therefore, it is not surprising that both species show low *Bd* infections in the wild [37]. Overall, our results show that vegetation cover influences *Bd* prevalence and infection intensity in temperate amphibian populations by modulating shade and associated microclimatic patterns, and that host community structure plays at most a minor role in our study system.

The best predictors of *Bd* prevalence and infection intensity were environmental variables associated with vegetation cover and microclimate (see Table 1). The amount of natural vegetation at the water line is strongly associated with the proportion of shade at the edge of the pond. Thus, the amount of canopy density directly affects air and water temperatures and serves as a proxy for the thermal conditions at frog basking sites. Canopy cover also regulates the availability of shallow, warm-water patches in which amphibians might reduce or clear *Bd* infections. Our path analyses confirm that canopy density indirectly controls both *Bd* prevalence and infection intensity through these changes in pond thermal profiles. We also found equal support from a model where canopy density directly affects *Bd* infection intensity (Fig. 3B). A study on *Bd* infection levels in red-spotted newts (*Notophthalmus viridescens*) in Pennsylvania showed that the proportion of leaf-litter and vegetation in the pond substrate correlated positively with *Bd* prevalence and infection intensity [23]. The authors propose that leaf-litter and emergent vegetation might increase *Bd* transmission by providing substrates for *Bd* growth. Alternatively, leaf-litter may be an indicator of canopy cover and total degree of shade, which are potentially the true drivers of higher *Bd* levels in natural forest habitats. Similarly, in Costa Rica and Australia, localities with little to no canopy cover may provide amphibians with a refuge from *Bd*-induced extinction [50], [51] presumably due to similar mechanisms related to micro-environmental controls. Two rainforest frog species that suffered severe declines in Australian rainforests have been rediscovered in open dry forest sites coexisting with high prevalence of *Bd* year round (above 69%) [50]. Our results corroborate these recent findings [23], [50], [51] and show that modulation of *Bd* infections by environmental factors is a general phenomenon, and that these environmental controls linking natural vegetation to *Bd* dynamics are similar in tropical and temperate amphibian communities.

Microclimate can affect amphibian disease risk in two ways by (i) regulating *Bd* growth and persistence in both host and the environment, and (ii) by changing host's ability to fight and clear *Bd* through thermoregulation. The growth rate of *Bd* in the laboratory is strongly temperature-dependent, with an optimum climatic envelope ranging between 17–25°C and reduced persistence at temperatures above 28°C [32]. Average maximum water temperatures across all our sampling ponds (i.e., 24.41°C±3.12 SD) fell near the upper threshold of this microclimatic envelope, indicating that *Bd* growth may have been limited during some periods in our highest-temperature ponds. In fact, in four of our ponds 40.85±14.42% SD of water temperature records were above 25°C during the study period. Our data closely match the association between *Bd* infections and temperature found by Raffel et al. [23], with low *Bd* infection intensities and prevalence at sites with average water temperatures above 23°C. In the State of Maine, USA, *Bd* infections were lower during the peak of the summer than in spring and fall, presumably because water temperatures often exceeded the optimal temperature range for *Bd* growth [37]. In the State of Arizona, *Bd*-infected frogs were largely absent from sites where spring water exceeded 25°C [52]. Additionally, laboratory experiments with an Australian *Bd* strain showed that pathogen growth *in vitro* is hampered by short exposures (i.e., one hour daily) to high temperatures that would typically be available to frogs at basking sites [53]. Some amphibians may rely on behavioural fever to fight *Bd* infections [54], therefore warmer and drier microclimates may decrease the odds of both *Bd* infection and transmission in open habitats [55]. At cooler temperatures, amphibian hosts may also lose the ability to mount antimicrobial responses, which translate in higher *Bd* loads [56]. In the Sierra Nevada, however, *Bd* infection intensity and frog survival were unrelated to water temperature [57], but the maximum temperature at the three focal high elevation sites rarely exceeded 25°C.

Our results indicate that the small-scale effects of vegetation and microclimate on our host-*Bd* system are larger than the effects imposed by density-dependent forces that typically predict prevalence and infection intensity in other temperate amphibians [58]–[60]. In a simple regression, capture rate positively predicted *Bd* infection intensity, potentially indicating density-dependent controls; however, this effect was marginal and capture rate was not a significant predictor of *Bd* when considered together with environmental factors in model selection. The potential effect of density on *Bd* was not linked to forest cover because we did not detect a significant effect of natural vegetation on host capture rate. Pathogen build-up to lethal infection intensities is more likely to occur in dense populations, under conditions that promote continuous reinfection of the hosts [59], [60]. Nonetheless, our focal species exhibited lower infection intensities than susceptible hosts in Sierra Nevada [58], [59], or persisted under host densities that might not trigger outbreaks. Future studies should investigate potential associations among vegetation type and long-term density-dependent factors of pathogen dynamics.

Earlier studies in both tropical and temperate zones have found ontogenetic differences in *Bd* susceptibility [22], [23]. In both cases, juveniles and sub-adults showed higher *Bd* prevalence and infection intensities. One potential explanation is that disease risk drops with age in response to host-acquired immunity, as repeated exposure to a given pathogen increases host resistance [28], [55]. In addition, a reorganization of host immune system occurs during metamorphosis, and postmetamorphic defenses may take some time to mature [28]. Although average body weight was in fact a negative predictor of *Bd* infection intensity when considered independently, this parameter became a weak predictor when considering other environmental variables in the analysis. This weak effect of host body weight on *Bd* infection coupled with the fact that vegetation cover had no influence on host capture rate is an indication that, in our study system, habitat change has a larger influence on pathogen fitness than on host fitness. This result suggests that our focal species is highly resistant to *Bd* regardless of microclimate and vegetation. In fact, *L. clamitans* persists with a local *Bd* strain in the laboratory within optimal *Bd* grow temperatures [61].

We have shown that disturbances to natural forest habitats reduce *Bd* infections in both temperate and tropical systems [6], which could mislead some decision makers to propose forest removal as an amphibian conservation strategy. However, habitat loss alone is the leading factor driving amphibian extinctions and declines worldwide [16], [17], [38], [62], thus intentional habitat disturbances will not serve as a strategy to prevent biodiversity loss due to wave-like *Bd* epidemics [63]. Fortunately, there are promising conservation strategies that do not include habitat alteration. For example, captive breeding of frogs with high immunogenetic *Bd* resistance or tolerance could be a useful tool for assisted reintroductions in the wild [64], and would be especially promising in areas of pristine rainforests where *Bd* is most prevalent. With the high rate of anthropogenic modification to temperate and tropical forests, understanding how vegetation cover and disease interact is critical for predicting *Bd* spread and developing appropriate management tools for wild populations. Our results indicate that species-specific *in situ* management strategies will need to consider fine-scale microclimatic factors to safeguard *Bd*-susceptible species with narrow geographic distributions [38] outside areas of climatic refugia [65].

## Supporting Information

Table S1 **Model selection for environmental and biological variables influencing** ***Batrachochytrium dendrobatidis*** **(** ***Bd*** **) prevalence and infection intensity in populations of** ***Lithobates clamitans*** **in the Adirondack region, New York, USA.**
(DOCX)
