# Supplementary material for: Disease Risk in Temperate Amphibian Populations Is Higher at Closed-Canopy Sites
Source: PLoS One. 2012 Oct 31;7(10):e48205. doi: 10.1371/journal.pone.0048205 (PMC3485156; doi:10.1371/journal.pone.0048205)
Supplement: Table S1 — Model selection for environmental and biological variables influencing Batrachochytrium dendrobatidis ( Bd ) prevalence and infection intensity in populations of Lithobates clamitans in the Adirondack region, New York, USA. (DOCX) [file pone.0048205.s001.docx]

**Table S1**. Model selection for environmental and biological variables influencing *Batrachochytrium dendrobatidis* (*Bd*) prevalence and infection intensity in populations of *Lithobates clamitans* in the Adirondack region, New York, USA.

| Model | AICc | No. of variables |
| --- | --- | --- |
| *Prevalence* |  |  |
| CANOPY | 75.607 | 1 |
| CANOPY, MAXTEMP | 81.512 | 2 |
| MAXTEMP | 82.334 | 1 |
| AVTEMP | 83.047 | 1 |
| CANOPY, AVTEMP | 83.666 | 2 |
| *Infection Intensity* |  |  |
| AVTEMP, NATVEG, AVTEMP*NATVEG | -17.690 | 3 |
| CANOPY | -10.657 | 1 |
| CANOPY, DIVERSITY | -5.951 | 2 |
| CANOPY, MAXTEMP | -3.032 | 2 |
| CANOPY, AVTEMP | -2.509 | 2 |

We ranked all possible models using Akaike Information Criterion (AICc); five best models are reported for each dataset. Best predictors are: canopy density (CANOPY), natural vegetation (NATVEG), water temperature - daily average (AVTEMP), water temperature - average maximum (MAXTEMP), and host community diversity (DIVERSITY).
